# Lesion detection in digital breast tomosynthesis: human reader experiments indicate no benefit from the integration of information from multiple planes

**DOI:** 10.1117/1.JMI.10.S1.S11915

**Published:** 2023-06-26

**Authors:** Christiana Balta, Ingrid Reiser, Mireille J. M. Broeders, Wouter J. H. Veldkamp, Ruben E. van Engen, Ioannis Sechopoulos

**Affiliations:** a Dutch Expert Centre for Screening (LRCB), Nijmegen, The Netherlands; b Radboud University Medical Center, Department of Medical Imaging, Nijmegen, The Netherlands; c The University of Chicago, Department of Radiology, Chicago, Illinois, United States; d Radboud University Medical Center, Department for Health Evidence, Nijmegen, The Netherlands; e Leiden University Medical Centre, Department of Radiology, Leiden, The Netherlands; f University of Twente, Technical Medicine Center, Enschede, The Netherlands

**Keywords:** digital breast tomosynthesis, image perception, observer performance, mammography, two-dimensional, three-dimensional

## Abstract

**Purpose:**

In digital breast tomosynthesis (DBT), radiologists need to review a stack of 20 to 80 tomosynthesis images, depending upon breast size. This causes a significant increase in reading time. However, it is currently unknown whether there is a perceptual benefit to viewing a mass in the 3D tomosynthesis volume. To answer this question, this study investigated whether adjacent lesion-containing planes provide additional information that aids lesion detection for DBT-like and breast CT-like (bCT) images.

**Method:**

Human reader detection performance was determined for low-contrast targets shown in a single tomosynthesis image at the center of the target (2D) or shown in the entire tomosynthesis image stack (3D). Using simulations, targets embedded in simulated breast backgrounds, and images were generated using a DBT-like (50 deg angular range) and a bCT-like (180 deg angular range) imaging geometry. Experiments were conducted with spherical and capsule-shaped targets. Eleven readers reviewed 1600 images in two-alternative forced-choice experiments. The area under the receiver operating characteristic curve (AUC) and reading time were computed for the 2D and 3D reading modes for the DBT and bCT imaging geometries and for both target shapes.

**Results:**

Spherical lesion detection was higher in 2D mode than in 3D, for both DBT- and bCT-like images (DBT: ${\mathrm{AUC}}_{2D}=0.790$, ${\mathrm{AUC}}_{3D}=0.735$, P=0.03; bCT: ${\mathrm{AUC}}_{2D}=0.869$, ${\mathrm{AUC}}_{3D}=0.716$, P<0.05), but equivalent for capsule-shaped signals (DBT: ${\mathrm{AUC}}_{2D}=0.891$, ${\mathrm{AUC}}_{3D}=0.915$, P=0.19; bCT: ${\mathrm{AUC}}_{2D}=0.854$, ${\mathrm{AUC}}_{3D}=0.847$, P=0.88). Average reading time was up to 134% higher for 3D viewing (P<0.05).

**Conclusions:**

For the detection of low-contrast lesions, there is no inherent visual perception benefit to reviewing the entire DBT or bCT stack. The findings of this study could have implications for the development of 2D synthetic mammograms: a single synthesized 2D image designed to include all lesions present in the volume might allow readers to maintain detection performance at a significantly reduced reading time.

## 1 Introduction

Digital breast tomosynthesis (DBT) has been shown to achieve improved breast cancer detection compared to digital mammography (DM).^1^^–^^4^ In DBT, several low-dose 2D projections of the compressed breast, acquired from different angles, are reconstructed into a pseudo-3D volume.^5^^,^^6^ This reduces tissue overlap and can result in improved detection performance. As a result, several screening trials have shown improved cancer detection rates and in settings where the initial recall was high, lower recall rates, with DBT compared to DM.^2^^,^^7^^–^^14^

As opposed to DM, DBT requires readers to review an image stack comprising all planes resulting from the reconstruction of the DBT volume instead of a single 2D image. The increased time required to review the entire image stack substantially increases radiologists’ workload, especially when reading breast cancer screening exams.^15^^–^^19^ Therefore, there is a need to alleviate the burden on radiologists through alternative reading strategies, such as artificial intelligence-based display and navigation. However, human reader strategies in volumetric medical image interpretation are not yet well understood.^20^

Several reader studies have investigated lesion detection and search in real and simulated computed tomography (CT) images, comparing reader performance in the entire image stack (3D reading mode) or in individual images of the image stack (2D reading mode). In 2006, Ellis et al. demonstrated that a stacked image display improved reading both in performance and speed, compared to viewing individual CT slices in a tiled display.^21^ In 2013, Drew et al. investigated radiologists’ search strategies in CT lung nodule detection and discovered that radiologists either inspected small regions in the image while scrolling back and forth (“drillers”) or inspected individual slices of the CT scan (“scanners”). The nodule detection rate of drillers was significantly higher than that of scanners. These findings might indicate that “drillers” were able to incorporate information from adjacent slices into their search process.^22^ Yu et al. compared human reader performance in a 2D and 3D reading mode using phantom images acquired on a diagnostic CT scanner. The phantom consisted of low-contrast spheres of multiple diameters embedded in a uniform background. In the 2D reading mode, images through the center of a sphere were displayed, while in 3D reading mode, readers were allowed to scroll through adjacent images. One reader improved the percentage of correct responses made out of the total number of responses by 0.02 when reading in 3D mode, which was statistically significant. The other two readers performed equally or better in 2D reading mode, but the differences were not statistically significant.^23^ Abbey et al. investigated human observer performance in 2D and 3D search tasks in simulated isotropic power-law and white noise volumes. Their 2D and 3D classification images indicated little use of information across multiple slices. For large signals embedded in power-law noise, the efficiency of human readers was greater in the 2D than in the 3D reading mode.^24^ Packard et al. investigated the impact of reconstructed slice thickness on signal known exactly (SKE) detection of simulated spherical lesions embedded in breast CT (bCT) patient images, for a range of lesion sizes. An optimal slice thickness was found, that increased with lesion size,^25^ indicating that useful information was present in adjacent regions of the breast volume in the vicinity of the lesion.

Little work has been done investigating 3D reading strategies in DBT. The DBT volume has unique properties compared to CT, namely the depth resolution in DBT is non-isotropic and depends on the DBT system scan range as well as the extent of the object in the direction of the X-ray tube scan.^26^ Our preliminary studies have indicated that human reader performance does not improve when information on adjacent is provided.^27^^,^^28^

Hence, in this work, we further explore the question of whether human readers are able to integrate information from adjacent planes in a 3D lesion detection task, for breast imaging both with DBT and bCT. Images for the reader studies were generated using a directional power-law model with embedded spherical and cylindrical targets and “imaged” with both DBT (50 deg scan angle) and bCT (180 deg scan angle).

## 2 Materials and Methods

The DBT images in this study were generated by projecting and reconstructing simulated breast tissue for a DBT-like system with an angular scan range of 50 deg and for a bCT (bCT)-like geometry with a scan range of 180 deg. The purpose of generating two image sets was to allow a comparison of reader performance in DBT, which has a non-isotropic depth resolution, to reader performance in CT images with isotropic resolution.

The reconstructed images were read by human readers, who reviewed the full 3D stack of planes by playing a movie (ciné mode) and the static 2D plane through the lesion center only. Signal detectability and reading times for both viewing modes were evaluated. Additional details on the methods used are given in the Supplementary Material.

### 2.1 Simulated Breast Tissue

Anthropomorphic breast backgrounds were generated using a validated breast tissue model that produces textures similar to those observed in mammography and DBT.^29^^–^^31^ This validated breast tissue model allows for generating realistic breast structures that exhibit directionality, similar to the structures embedded in real breast parenchyma, which are typically directed towards the nipple.^32^ Realistic breast backgrounds were generated with different realizations of directionality orientation and strength based on measurements from clinical mammograms in earlier work of Reiser et al.^33^

### 2.2 Simulated Breast Lesions

**Fig. 1:**
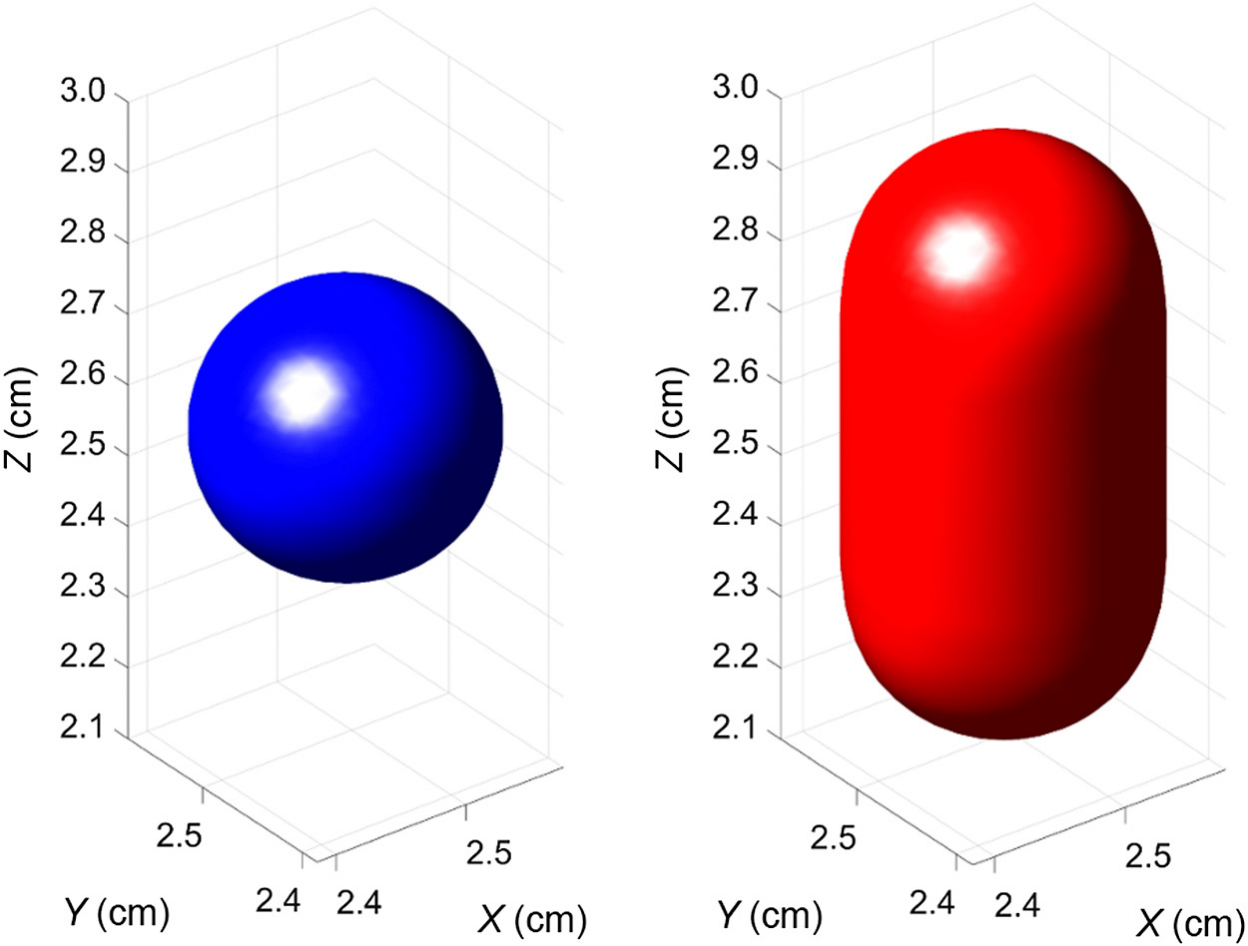
3D volumetric rendering of the simulated signals. The axis measurements provide the actual position of the lesions in the simulated background cubes.

As a first step, two simplistic lesion shapes were investigated, a 4 mm diameter spherical signal and an elongated signal created as a capsule with a prolate symmetry. The capsule-shaped signal was included to produce a target that persists for a longer time, as it is present in a larger number of image planes. The capsule was formed by adding two 4 mm diameter half-spheres to the ends of a cylinder, to make a capsule with an overall length of 8 mm (Fig. 1). The capsule was aligned perpendicular to the reconstructed images to extend across more planes than the sphere. Edges of both shapes were smoothed by filtering the signals with a Gaussian function with a standard deviation of σ=0.3  mm.

### 2.3 Image Simulations

The simulated backgrounds and lesions were numerically projected and reconstructed using the ASTRA toolbox (iMinds-Vision Lab, University of Antwerp, Belgium).^34^ The image simulation assumed a parallel X-ray beam geometry where the X-ray source and detector follow a predefined circular trajectory around the object. To be able to discriminate any possible effects of the incomplete angular sampling due to the limited angle nature of DBT, two imaging geometries were studied; a DBT-like geometry with an angular scan range of 50 deg and 25 projections and a bCT-like geometry with a range of 180 deg and 180 projections.

The volumes were reconstructed using filtered-back projection with a Hanning window. To simulate DBT images that better reflect the asymmetric voxel sizes of clinical images, binning was performed along the z-axis by averaging planes in groups of five, resulting in images at a 1 mm spacing, with an in-plane pixel size of $0.2\times 0.2\;{\mathrm{mm}}^{2}$. The final 3D array size was $256\times 256\times 51\;{\mathrm{pixels}}^{3}$. This array size coincides with the image size used in the reader study.

From each reconstructed image volume, a single 2D image and a 3D image stack were extracted. The 3D image stack for bCT and DBT included 26 planes in total. To simulate lesions, 3D signals were inserted at the center of the 3D image stack. The 2D image was the plane through the lesion center, extracted from the 3D stack. Thus, the signal amplitude was equal in 2D or 3D. Signal amplitude was set so that readers would achieve ∼80% correct responses when reviewing the 2D image only, based on the results of a small pilot reader study.

### 2.4 Reader Study

The 3D viewing mode was a ciné loop at a fixed speed of 10 frames per second. The readers could not control the scrolling speed, but they could go over the loop as many times as needed to arrive at a decision. This was done to ensure that the readers would interpret the whole 3D stack of images rather than immediately scroll to the lesion center, which would mean they are performing a 2D detection task while neglecting the adjacent image planes.

**Fig. 2:**
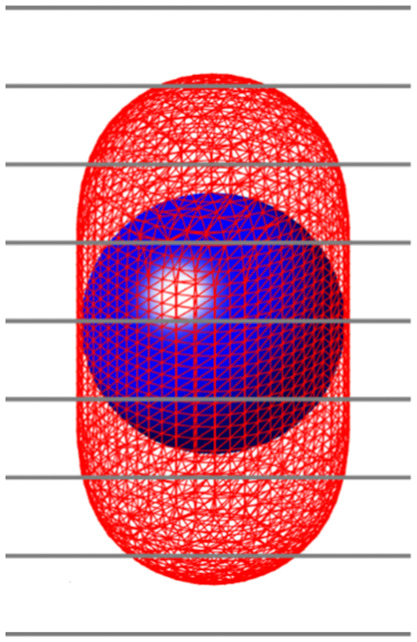
Example of the planes spanning over the sphere and capsule. Due to their low contrast, the lesions were mostly visible in the central plane that intersected each corresponding lesion.

**Fig. 3:**
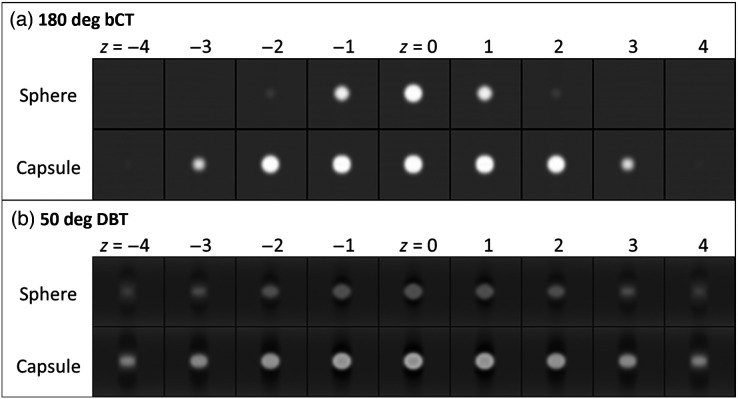
Visualization of the sphere and capsule in planes above and below the plane through the target center (z=0) for (a) 180 deg bCT and (b) 50 deg DBT. Plane spacing is 1 mm. Only the center region of the image array is shown.

The visualization of signals was different for the spherical and capsule-like signal shapes. For the sphere, the diameter of the signal visualized in planes above or below the center was smaller, while for the capsule, the signal persisted at the same diameter in about five adjacent image planes (Fig. 2). This repetition of the signal across multiple planes of a 3D volume was the reason for also performing this study with capsule-shaped signals in addition to the spherical ones. Figure 3 shows reconstructions of the spherical and capsule signals across multiple adjacent planes. For the bCT-like geometry with isotropic depth resolution, the signals are visualized in planes consistent with Fig. 2. For the DBT geometry, both the sphere and capsule is still seen perceived in planes that are further from the in-focus plane, which is caused by the poor depth resolution of DBT due to the limited angle scan.

Signal amplitude was set so that readers achieved ∼80% correct responses when viewing the 2D image through the center of the sphere, and was determined in pilot experiments.

**Fig. 4:**
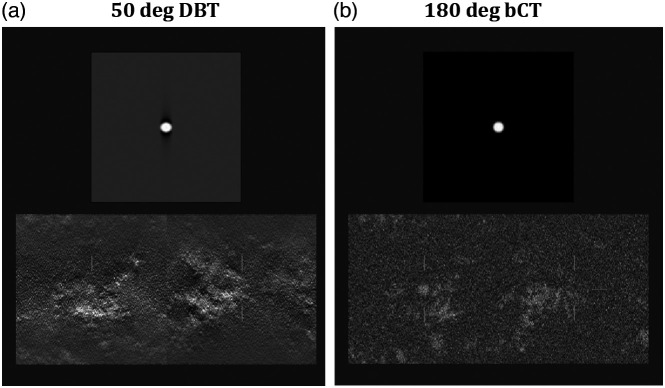
3D viewing mode of the capsule-like lesion (signal is on the left image): (a) 50 deg DBT images and (b) 180 deg bCT-like images.

**Fig. 5:**
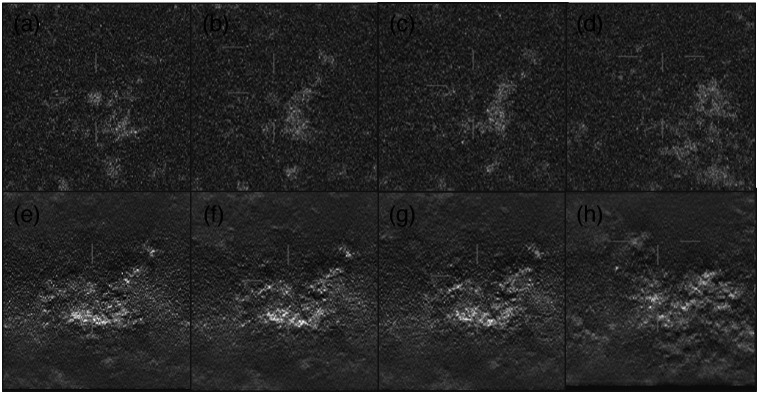
Visualization of the sphere embedded into backgrounds in multiple adjacent planes. Plane spacing is 1 mm. (a)–(d) 180 deg bCT-like images (e) and (f) 50 deg DBT images.

Low-contrast signal detection performance was measured through two-alternative forced-choice (2AFC) experiments. In each trial, a signal-absent and a signal-present image were shown, and the reader was instructed to select the signal-present image. The readers knew that the lesions had a circular cross-section in the plane of view, but were not informed that one of the signals, the capsule, was elongated along the depth direction. There was no time limit to arrive at a decision, and reading times were recorded for each image pair. Horizontal and vertical location cues were displayed on both signal-present and signal-absent images (Fig. 4). In the cine-loop display, the horizontal cues indicated the depth of the displayed plane within the volume, such that the horizontal and vertical cues formed a cross at the center of the volume. Figure 5 shows the succession of a sphere embedded into backgrounds both for the bCT-like and the DBT imaging geometries.

One hundred trials were performed for each experimental condition. Each trial showed two different breast backgrounds, which required generation of 200 breast volumes. There were a total of eight experimental conditions (sphere or capsule, 2D or 3D, 50 deg, or 180 deg angular range). 11 medical physicists participated in the reader study with experience in DM and DBT varying from 1 to 20 years and 1 to 15 years, respectively.

The study was performed in a reading room with low ambient light, similar to the clinical conditions. The study was performed using a DICOM-calibrated 12 MP high-luminance DM/DBT monitor (Coronis Uniti (MDMC-12133), Barco, Belgium) using an in-house developed 2AFC software.^28^

### 2.5 Statistical Analysis

Multiple-reader, multiple-case (MRMC) analysis of all reader responses was performed using iMRMC software (iMRMC version 4.0, Division of Imaging, Diagnostics, and Software Reliability, OSEL/CDRH/FDA, Silver Spring, Maryland, United States).^35^^–^^37^

The reader-specific area under the receiver operating characteristics curve (AUC) values and their 95% confidence intervals were computed following U-statistics using the one-shot method of Gallas.^35^ The reader-averaged AUC was calculated by averaging the reader-specific, non-parametric AUC. A P-value of 0.05 or less was considered to indicate a statistically significant difference.

Reading times, defined as the time spent per trial (or image pair), were compared between 3D and 2D viewing using two-sample unpaired t-tests. P<0.05 was considered indicative of a statistically significant difference. Reading time outliers, defined as values extending above 2 standard deviations of the mean under a given condition, were removed since the reading could have been interrupted.

## 3 Results

### 3.1 Detection Performance

Reader-averaged AUC for 2D and 3D viewing modes are shown in Tables 1 and 2 for angular ranges of 50 deg and 180 deg, respectively. For the sphere detection in DBT images, the readers had a higher ${\mathrm{AUC}}_{2D}$ than an ${\mathrm{AUC}}_{3D}$ with a difference of 0.055 (P<0.05). For capsule detection in DBT images, the difference between average ${\mathrm{AUC}}_{2D}$ and average ${\mathrm{AUC}}_{3D}$ was not statistically significant (P=0.19) (Table 1).

**Table 1:** Mean AUC for all readers and difference in 2D and 3D viewing mode for the 50 deg angular range DBT images.

Signal	Mean ${\mathrm{AUC}}_{2D}$	Mean ${\mathrm{AUC}}_{3D}$	Difference	p-value
Sphere	0.790	0.735	0.055	<0.05
Capsule	0.891	0.915	−0.024	0.19

**Table 2:** Mean AUC of the readers and difference in 2D and 3D viewing mode for the 180 deg angular range bCT-like images.

Signal	Mean ${\mathrm{AUC}}_{2D}$	Mean ${\mathrm{AUC}}_{3D}$	Difference	P-value
Sphere	0.869	0.716	0.153	<0.05
Capsule	0.854	0.847	0.006	0.88

Table 2 shows the mean AUC of the readers in the bCT-like images. For the sphere detection, the readers had a higher ${\mathrm{AUC}}_{2D}$ than an ${\mathrm{AUC}}_{3D}$ with a difference of 0.153 (P<0.05). Regarding the detection of the capsule, readers’ ${\mathrm{AUC}}_{2D}$ and ${\mathrm{AUC}}_{3D}$ were not found to be statistically different (P=0.88).

**Fig. 6:**
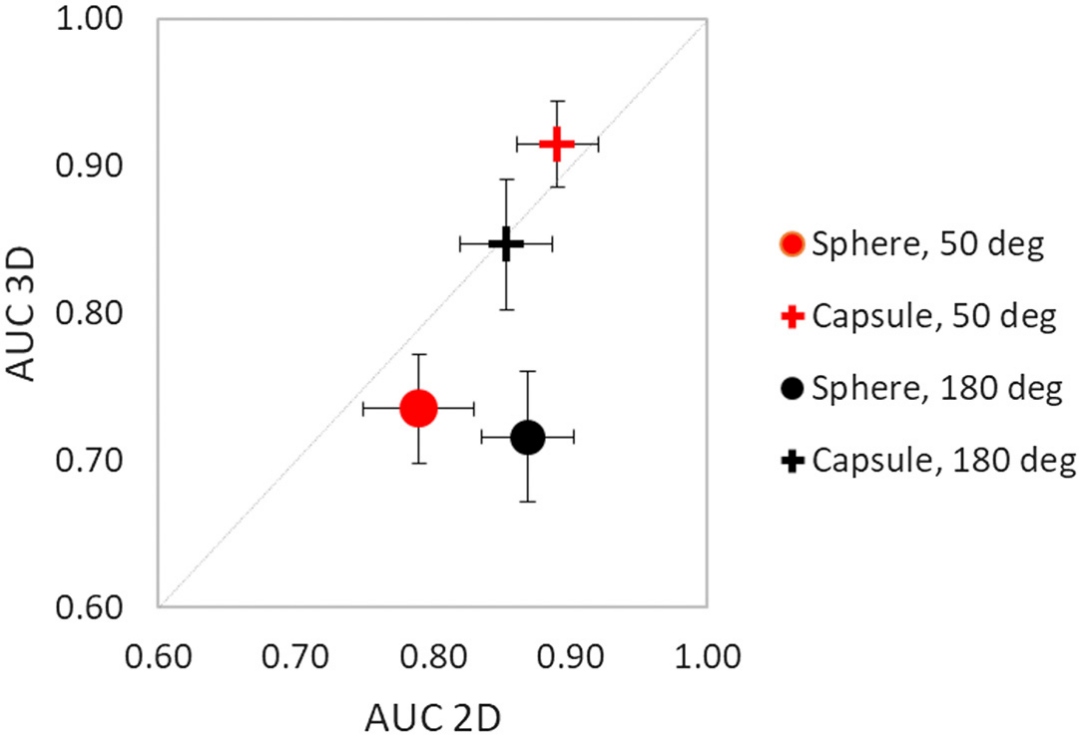
Comparison of reader-averaged AUC results in 2D and 3D reading modes for all experimental conditions. The total length of error bars is 2 standard deviations.

The reader-averaged AUC results for the 50 deg and the 180 deg angular range for the detection of spheres and capsules are shown in Fig. 6. The 3D sphere detection was the most difficult task for both angular ranges resulting in the lowest AUC values. The lowest average AUC value of 0.716 was observed in the 180 deg images followed by the average AUC of 0.735 in the 50 deg images. All individual AUC values of all readers for all conditions are given in the Supplementary Material.

### 3.2 Reading Time

**Fig. 7:**
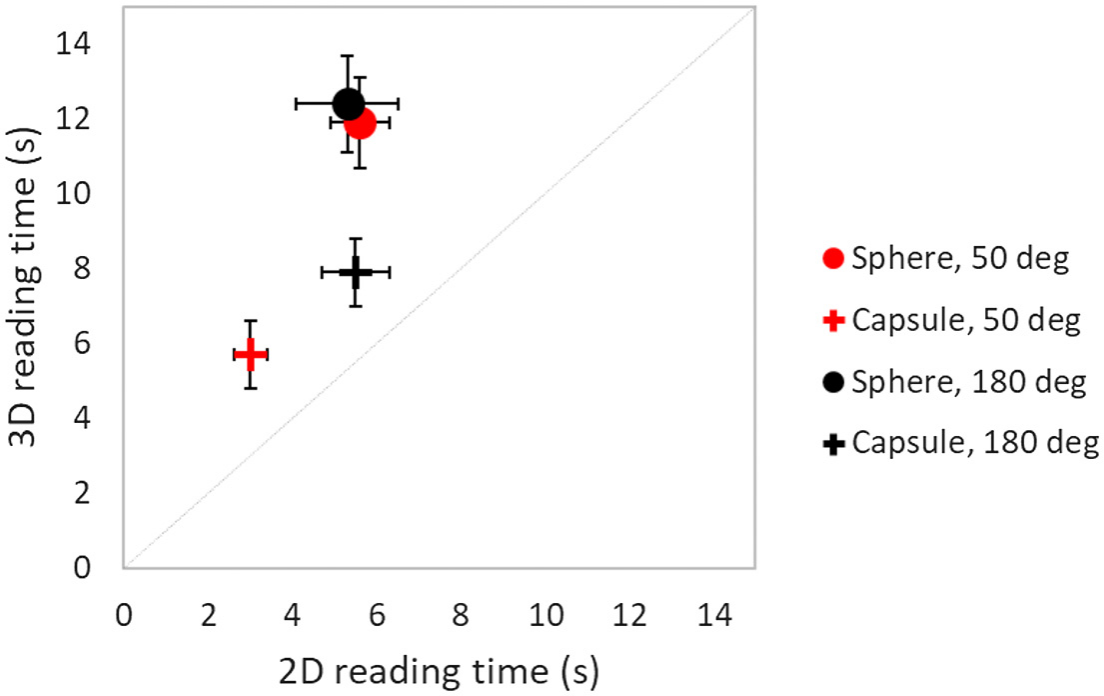
Comparison of reader-averaged reading times in 2D and 3D reading modes for all experimental conditions. The total length of error bars is 2 standard deviations.

As shown in Fig. 7 and Tables 3 and 4, the average reading time per trial was up to 134% higher for 3D than for 2D viewing. The difference in reading time was higher for the spherical target than for the capsule, as shown in Tables 3 and 4. Also, the conditions with the lowest AUCs had the longest reading times. For all signals (sphere and capsule) reading time outliers were found to be 20% out of the total number of 2D and 29% out of the total number of 3D cases, respectively.

**Table 3:** Mean reading times for the 50 deg DBT images.

Signal	Mean reading time 2D (s)	Outliers excluded (%)	Mean reading time 3D (s)	Outliers excluded (%)	Mean reading time increase (%)	P-value
Sphere	5.6	4.7	11.9	9.5	112.5	<0.05
Capsule	3.0	3.6	5.7	4.4	90	<0.05

**Table 4:** Mean reading times for the 180 deg bCT-like images.

Signal	Mean reading time 2D (s)	Outliers excluded (%)	Mean reading time 3D (s)	Outliers excluded (%)	Mean reading time increase (%)	P-value
Sphere	5.3	7.2	12.4	4.6	134.0	<0.05
Capsule	5.5	5.3	7.9	10.7	43.6	<0.05

## 4 Discussion

This work is the first step to forming an understanding of viewing strategies for 3D medical images and particularly whether humans integrate signals over adjacent planes. The main findings are: (1) for spherical signals, 3D viewing reduced observers’ detection performance, (2) for elongated spherical signals 3D viewing was equal to 2D viewing, and (3) these findings were similar in images of two different acquisition angular ranges investigated simulating DBT and bCT.

Reader performance for detecting the spherical or capsule-shaped targets was equal in the 2D CT mode, which is expected since CT images are true cross-sectional images. On the other hand, in DBT, the 2D detection performance of the capsule-shaped target was greater than that of the spherical-shaped target, which is also in agreement with the more projection-like nature of DBT imaging, which is considered a “quasi-3D” but not a true 3D image volume.

The lack of integration across multiple slices provides an explanation for the lower detection performance of readers demonstrated in the 3D tasks. With the target being present in a random location in the image, Abbey et al.^24^ used localization efficiency to assess the extent of 3D image information that is being processed by human observers. Even though a basis image formation model was used, their findings are consistent with ours. Readers were more efficient in the 2D task than the 3D task for a 4 mm diameter target, even though it spanned multiple planes.^24^

Since 3D viewing of volume images does not seem to benefit the readers, this finding is of relevance for reducing the DBT reading times by the use of a 2D image.

Synthetic mammograms (SM), i.e., planar DM-like images generated from the DBT data, has been investigated for their potential for providing an overview of the DBT volume. Thus far, the development and evaluation of SM has focused on eliminating the conventional 2D mammogram, which is acquired along with the DBT during breast cancer screening.^38^^–^^41^

Our findings suggest that an “ideal SM,” that consisted of all suspicious lesions present in the DBT volume, could eliminate the need for reading the DBT volume altogether, since no additional information is contained in DBT planes outside of the focus of a lesion. Such an “ideal SM” might be generated by utilizing artificial intelligence (AI) approaches^42^^–^^44^ to detect suspicious lesions present in the DBT volume and then fitting a minimally bent plane through the locations of suspicious findings^45^ or by blending the suspicious findings from the various DBT planes onto one SM plane.^46^ In breast cancer screening, an AI-generated 2D SM has resulted in better lesion detection performance and in faster reading time compared to DM^45^ with non-inferiority of radiologist interpretation performance.^46^

The study has limitations. First, images consisted of simulated signals and backgrounds. While the backgrounds have been validated to be realistic in earlier studies,^30^ the use of simplistic lesion-like signals may not reflect the typical clinical findings. Since our motivation is not specific to any particular clinical finding but to investigate whether the presence, or absence, of a signal triggers the human visual system differently in 2D than in 3D viewing, our approach is based on simulated signals. Also, having a perfectly aligned capsule-like signal in the *z*-direction might be clinically impossible, but it was deliberately used to assess the impact of the lower depth resolution in DBT, which causes signals to be “replicated” or “blurred” into adjacent image planes—whereas the capsule-like signal extended over multiple image planes both in DBT and CT.^6^^,^^47^ Another limitation of this study is that we investigated a signal-known-exactly detection task, which is different from the clinical reading where radiologists typically perform a search of an unknown lesion. However, we presume that if there is any difference between the 2D and 3D reading conditions this would have been projected similarly on a signal-known-exactly task and a clinical reading task. In the future, with the advent of technology in terms of SM image generation from DBT, clinical SM images (optimized to include all suspicious regions in the composite image) should be investigated and benchmarked with our results.

These preliminary results are not intended to provide reading strategies. Future technological advancements are required to tackle the unnecessary image information and excessive “scrolling” involved in DBT. This would have a clinical impact in situations where reading time reduction and sensitivity are of concern, such as in the case of DBT screening. The insights gained in this study and perhaps on follow-up studies along the lines discussed above could provide valuable information when developing new alternative reading strategies for tomographic and pseudo-tomographic imaging.

## 5 Conclusion

In this human reader study with stylized low-contrast lesions embedded in simulated breast backgrounds, no inherent visual perception benefit to reviewing the entire DBT or bCT stack was found. This suggests that lesion detection performance might be maintained in an ideal 2D SM that includes all lesions present in the DBT image volume.
