# Supplementary material for: Lesion detection in digital breast tomosynthesis: human reader experiments indicate no benefit from the integration of information from multiple planes
Source: J Med Imaging (Bellingham). 2023 Jun 26;10(Suppl 1):S11915. doi: 10.1117/1.JMI.10.S1.S11915 (PMC10292860; doi:10.1117/1.JMI.10.S1.S11915)
Supplement: Supplementary file 1 [file JMI_010_S11915_SD001.pdf]

S. Supplemental Materials

S1. Methods: Image simulations

The simulated breast tissue volumes were based on a power-law texture, as an approximation of anatomical variability based on previous publications. Cubes with a side length of 5.12 cm, and with isotropic voxels of 0.2 mm^3. The breast-like structures generated in this study displayed directionality towards the nipple with variable orientation angles (θ) from a random distribution with $\bar{\theta}$ =4° and σ_θ=23°. The strength of this directionality (q) was also variable with $\bar{q} = 1.875$ and $\sigma_q = 0.47$. The image reconstructions were performed at the phantom's native resolution $(0.2\ mm)^3$ in a MATLAB interface and were accelerated using graphics processing unit computing.

Quantum noise in each detector bin was introduced by a transformation from the transmission to the number of photons, which was then converted to random Poisson numbers. The noise level corresponded to that of an average glandular dose of 1.5 mGy for both acquisition geometries. The air kerma for the total of all projections was 4.385 mGy. The number of incident photons per pixel, N_0, was calculated from Boone et al. [39] for a 5 cm thick and 50% glandular breast assuming mono-energetic x-rays at 20 keV and photon fluence per unit exposure of $5 \cdot 10^4 \frac{photons}{mm^2 mR}$.

The resulting noisy photon counts n *are transformed back to obtain noisy projection data* by taking the negative logarithm*:*

$$n' = -\log \frac{n}{N_0}$$

The noisy sinogram n', was used as input to the reconstruction.

Detector blur was simulated by convolving the photon distribution with a Gaussian of width σ_g = 0.5 pixels (27).

S2. Methods: Reader study

While the order of 2D or 3D experiments differed per observer, within each experiment, the order of the cases in each experiment and the signal location ground truth were the same for all observers. Each reader performed 4 experiments for one angular span in one day. The readers were instructed to take a break after 2 experiments to prevent fatigue.

The observers' age varied from 25 to 61 years old with a median of 31 years old. Before each experiment, a training session was given for each reader that involved 10 pairs with signals of higher contrast and 6 pairs with similar signal contrast to the experiment they were about to perform. The readers were provided with feedback on their performance after the end of each training session. During the actual experiment, no feedback was given to the readers and readers did not have the opportunity to go back on their decisions.

S3. Results: AUC values of all readers

Individual readers' AUC for the task of detecting a low-contrast signal is shown in Supplemental Figure S1 (DBT images) and Figure S2 (bCT-like images). For all signal shapes and angular ranges, AUC for the 3D viewing mode was never statistically significantly higher than AUC for the 2D viewing mode.

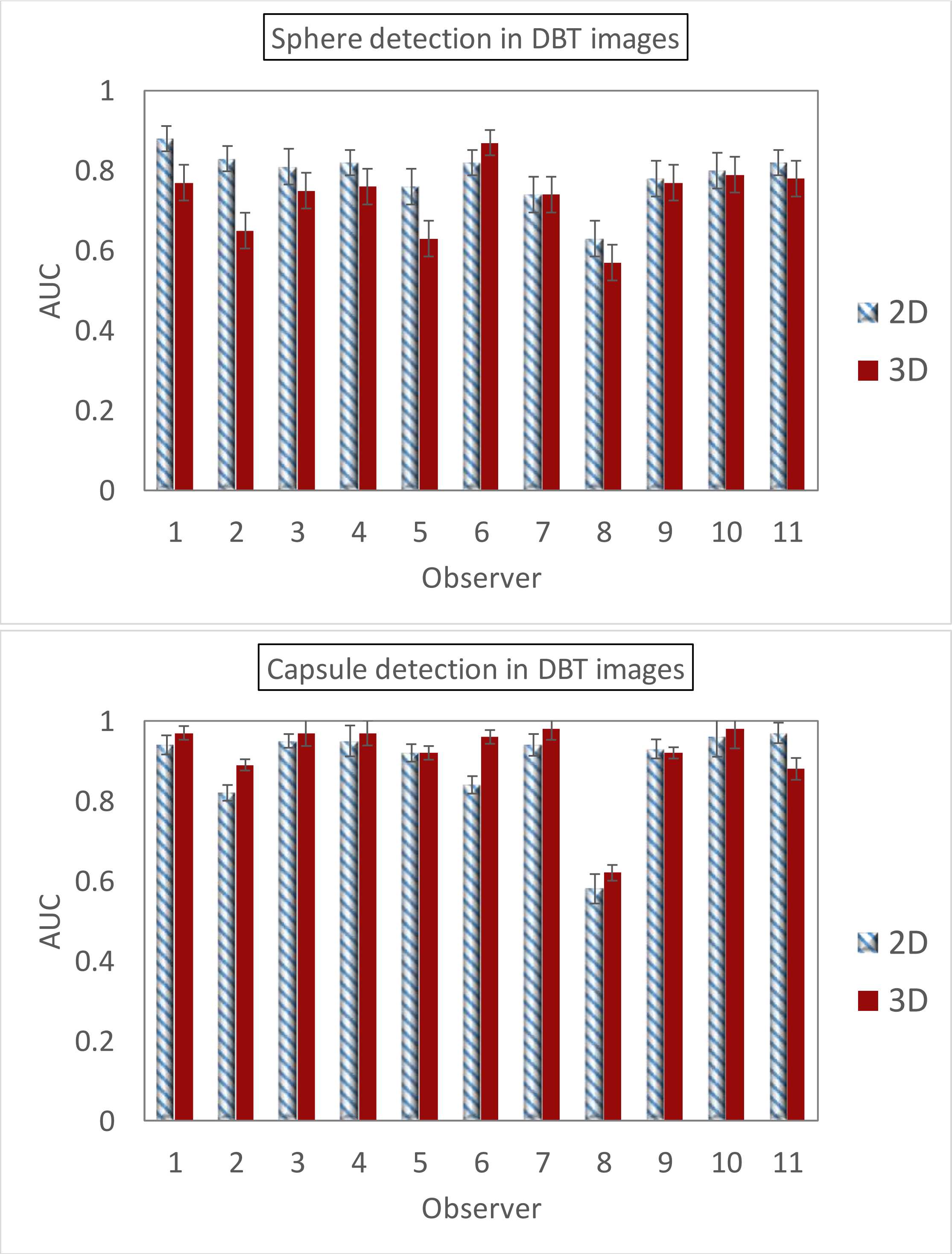

Figure S1: Performance of 11 readers in the task of detecting a sphere (top) or a capsule (bottom) in a DBT volume. Results for reading in 2D (blue) and 3D (red) are shown. The total length of error bars are 2 standard deviations.

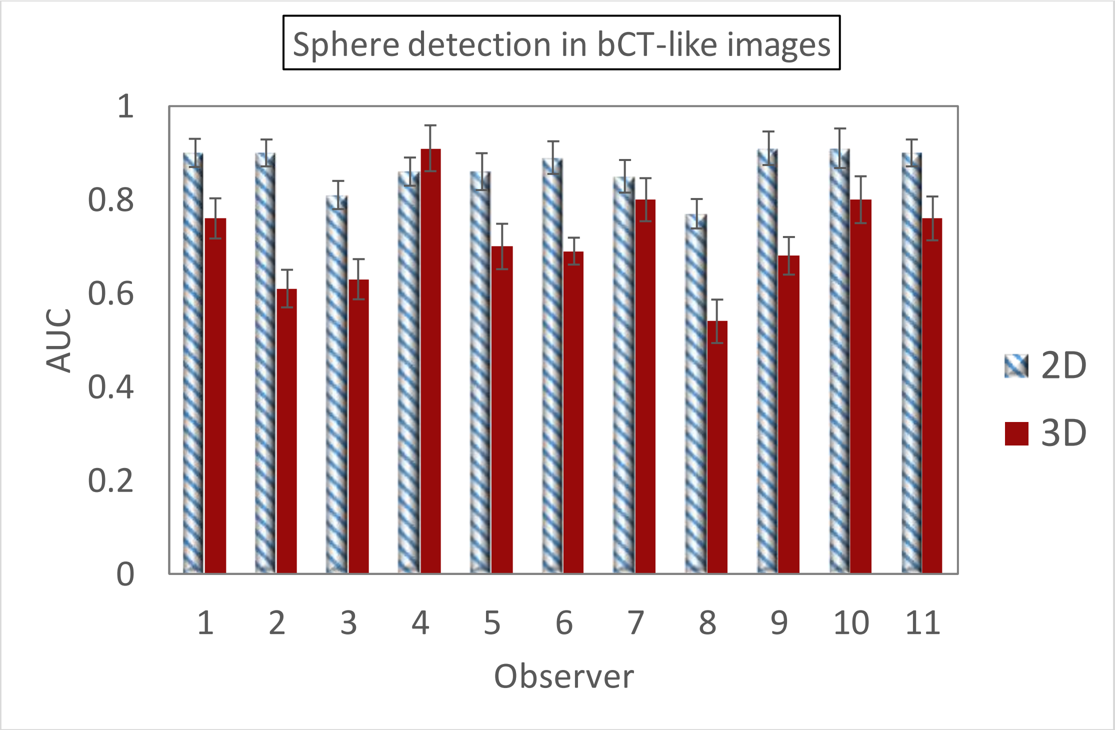

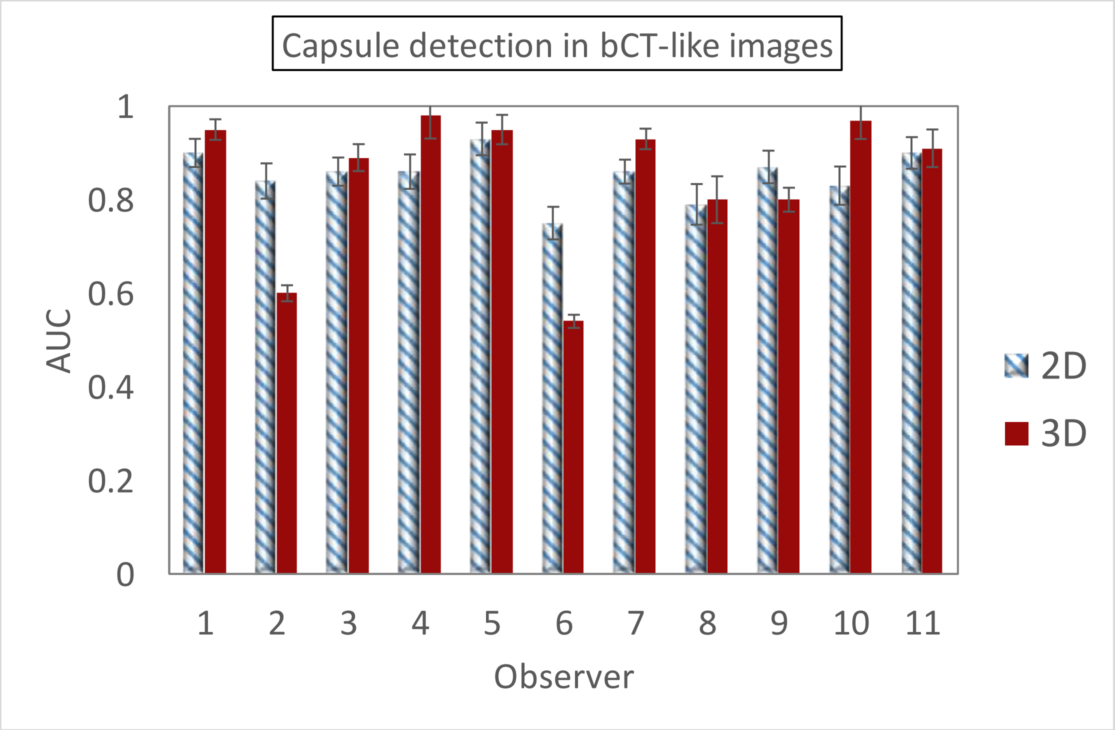

Figure S2: Performance of 11 readers in the task of detecting a sphere (top) or a capsule (bottom) in a bCT-like volume. Results for reading in 2D (blue) and 3D (red) are shown. The total length of error bars are 2 standard deviations.
